# A Comparative Study of Structural and Metabolic Brain Networks in Patients With Mild Cognitive Impairment

**DOI:** 10.3389/fnagi.2021.774607

**Published:** 2021-12-06

**Authors:** Cuibai Wei, Shuting Gong, Qi Zou, Wei Zhang, Xuechun Kang, Xinliang Lu, Yufei Chen, Yuting Yang, Wei Wang, Longfei Jia, Jihui Lyu, Baoci Shan

**Affiliations:** ^1^Innovation Center for Neurological Disorders and Department of Neurology, Xuanwu Hospital, Capital Medical University, National Clinical Research Center for Geriatric Diseases, Beijing, China; ^2^Center of Alzheimer’s Disease, Beijing Institute for Brain Disorders, Beijing Key Laboratory of Geriatric Cognitive Disorders, Neurodegenerative Laboratory of Ministry of Education of the People’s Republic of China, Beijing, China; ^3^School of Biological Science and Medical Engineering, Beihang University, Beijing, China; ^4^College of Integrated Traditional Chinese and Western Medicine, Changchun University of Chinese Medicine, Changchun, China; ^5^Institute of High Energy Physics, Chinese Academy of Sciences, Neurodegenerative Laboratory of Ministry of Education of the People’s Republic of China, Beijing, China; ^6^Center for Cognitive Disorders, Beijing Geriatric Hospital, Beijing, China

**Keywords:** mild cognitive impairment, brain network, structure, metabolism, brain regions

## Abstract

**Background:** Changes in the metabolic and structural brain networks in mild cognitive impairment (MCI) have been widely researched. However, few studies have compared the differences in the topological properties of the metabolic and structural brain networks in patients with MCI.

**Methods:** We analyzedmagnetic resonance imaging (MRI) and fluoro-deoxyglucose positron emission tomography (FDG-PET) data of 137 patients with MCI and 80 healthy controls (HCs). The HC group data comes from the Alzheimer’s Disease Neuroimaging Initiative (ADNI) database. The permutation test was used to compare the network parameters (characteristic path length, clustering coefficient, local efficiency, and global efficiency) between the two groups. Partial Pearson’s correlation analysis was used to calculate the correlations of the changes in gray matter volume and glucose intake in the key brain regions in MCI with the Alzheimer’s Disease Assessment Scale-Cognitive (ADAS-cog) sub-item scores.

**Results:** Significant changes in the brain network parameters (longer characteristic path length, larger clustering coefficient, and lower local efficiency and global efficiency) were greater in the structural network than in the metabolic network (longer characteristic path length) in MCI patients than in HCs. We obtained the key brain regions (left globus pallidus, right calcarine fissure and its surrounding cortex, left lingual gyrus) by scanning the hubs. The volume of gray matter atrophy in the left globus pallidus was significantly positively correlated with comprehension of spoken language (*p* = 0.024) and word-finding difficulty in spontaneous speech item scores (*p* = 0.007) in the ADAS-cog. Glucose intake in the three key brain regions was significantly negatively correlated with remembering test instructions items in ADAS-cog (*p* = 0.020, *p* = 0.014, and *p* = 0.008, respectively).

**Conclusion:** Structural brain networks showed more changes than metabolic brain networks in patients with MCI. Some brain regions with significant changes in betweenness centrality in both structural and metabolic networks were associated with MCI.

## Introduction

Alzheimer’s disease (AD) is a common neurodegenerative disorder, which is the leading cause of dementia (Alzheimer’s Association, 2016). Mild cognitive impairment (MCI) is an intermediate state between normal aging and dementia (Yao et al., 2010; Liu et al., 2012), and approximately 10–15% patients with MCI progress to AD every year (Petersen et al., 1997; Davatzikos et al., 2011). Therefore, the pathological mechanisms underlying MCI should be explored. Additionally, effective interventions in the MCI phase may considerably reduce the incidence of AD.

Magnetic resonance imaging (MRI) and 18F-labeledfluoro-deoxyglucose positron emission tomography (FDG-PET) are common neuroimaging modalities, whichrespectively reflect the glucose metabolism in different brain regions and analyze the characteristic features of brain atrophy such as whole brain volume, localized brain areas, cortical thickness, and curvature in a reliable manner (Son et al., 2015; Hojjati et al., 2019). Currently, MRI and PET findings, such as hippocampal gray matter atrophy and hypometabolism in the posterior cingulate cortex and temporoparietal cortex, pertaining to individual brain regions have been shown to serve as *in vivo* imaging markers for the diagnosis of AD (Mutlu et al., 2016). However, the function of the brain is not determined only by a single brain region, but by a series of interactions among brain regions (Mutlu et al., 2016). The emergence of brain networks has provided a new method for understanding the connections among cerebral regions contributing to the potential findings of AD that can help in diagnosis, predicting disease progression, and exploring pathogenesis.

The current focus is on research regarding AD from the perspective of brain networks. Brain networks provide biomarkers to distinguish between normal cognition and MCI. The importance of nodal graph measurements as markers in the early diagnosis of AD has been demonstrated (Xu et al., 2020). The right Crus II of the cerebellar hemisphere and fusiform gyrus could be the potential diagnostic biomarkers for MCI (Zhang et al., 2020). Moreover, brain networks can predict the progression of MCI to AD because of their close relationship with the course of the disease in the AD continuum (Sun et al., 2018; Zheng et al., 2019). The emergence of brain networks has also provided a new perspective for explaining the pathogenesis of AD. He et al. (2008) established the first structural brain network model of AD in 2008 and found that the cortical network and regional centrality of patients with AD were destroyed, which proved that the pathological changes in AD were associated with the destruction of large-scale brain networks (He et al., 2008). Similar to the results pertaining to brain networks, the topological properties of brain network building have been reported to be damaged, as evident from MRI, FDG-PET, and resting state-functional MRI (rs-fMRI) data (Yao et al., 2010; Sanabria-Diaz et al., 2013; Sun et al., 2014). The degree of variations in specific network parameters [e.g., small-world properties, characteristic path length (*L*), clustering coefficient (*C*), local efficiency (*E*_*loc*_), and global efficiency (*E*_*glob*_)] of patients with MCI lies between that of healthy individuals and patients with AD, representing a continuity from aging to AD.However, to describe the complex pathological mechanisms underlying AD, the information provided by the network built by these single imaging modes is limited.

In contrast to the shortage of single imaging modes, studies assessing the multiple modes of brain network can easily clarify the pathological status by comparing the differences and internal relationships among two or more modal information. A previous study comparing brain networks based on rs-fMRI and diffusion tensor imaging (DTI) data reported that there was no one-to-one relationship between functional and structural connection strengths of different brain regions in MCI networks (Sun et al., 2014). The asynchrony in the damage between the two brain networks was shown in Palesi’s research, in which the functional network changed in MCI, the early state of AD, before the destruction of the structural network. rs-fMRI-based functional connectivity is significantly altered in AD and MCI, whereas DTI-based structural connectivity is shifted significantly only in AD (Palesi et al., 2016). However, another study using MCI data showed conflicting results (Filippi et al., 2020), which may be due to the difference in DTI reconstruction methods. At present, studies that analyze the difference between metabolic networks and structural modes in MCI use data collected from DTI and fMRI scans. Little is known about the associations between MRI and FDG-PET networks in the MCI stage.

In this study, it is hypothesized that the shape of structural brain network damaged differ from the metabolic network under AD pathology, but that the damage in two brain networks was related. We compared the topological properties of different modes in MCI and observed the differences in topological parameters of structural and metabolic networks between patients with MCI and healthy controls (HCs). The key brain regions of networks were determined by screening important hub nodes with significant changes in betweenness centrality in both the structural and metabolic networks. Finally, we analyzed the potential correlation between key brain regions and cognitive function. Our research will help in understanding the metabolic mechanisms associated with the structural disconnection during MCI, and show the brain areas that may be affected by the pathogenesis of AD in the brain network.

## Materials and Methods

### Participants

We recruited 137 patients diagnosed with MCI from 25 hospitals in China and 80 healthy subjects from the Alzheimer’s Disease Neuroimaging Initiative (ADNI) database^1^ to serve as HCs. The ADNI was launched in 2003 as a public-private partnership led by principal investigator Michael W. Weiner, MD. The primary goal of ADNI has been to assess whether serial MRI, PET, other biological markers, and clinical and neuropsychological assessment can be combined to assess the progression of MCI to early AD.

Patients included in the study were hospitalized or out-patients with MCI aged 50–85 years. Referring to the 2011 clinical MCI diagnostic criteria by the National Institute of Aging and Alzheimer’s Disease Association (NIA-AA), patients were diagnosed according to a comprehensive assessment including clinical history, neurological examination, and neuropsychological tests. Inclusion criteria were patients who were right-handed, who were hospitalized or out-patients aged between 50 and 85 years, with diagnosis of probable MCI according to established criteria (McKhann et al., 2011), with Mini-Mental State Examination (MMSE) scores of 20–26 (including 20 and 26),Clinical Dementia Rating (CDR)score of 0.5, and who could professionally communicate in Chinese (non-illiterate). Exclusion criteria included patients with a diagnosis of dementia, focal or diffuse brain damage, severe leukoencephalopathy, Fazekas scores ≥ 3, consciousness disorders, severe aphasia or physical disability that could interfere with neuropsychological examination, history of alcoholism, and history of drug addiction. Participants of the ADNI were included in this study if they met the following criteria: age between 50 and 80 years, non-depression, non-MCI, and non-dementia, with an MMSE score of 24–30 and CDR score near zero. All participants (or their caregivers) provided written informed consent prior to study inclusion.

### Neuropsychological Assessment

All patients underwent neuropsychological evaluations, including MMSE (Tombaugh and McIntyre, 1992) and Alzheimer’s Disease Assessment Scale-Cognitive (ADAS-cog) 11 (Rosen et al., 1984). MMSE is the favored assessment method for dementia screening and is performed by professional neuropsychologists. It allows the assessment of seven cognitive domains, including time and site orientation, comprehension, language, immediate and delayed memory, attention, visual space, and calculation, with a maximum score of 30. ADAS-cog is one of the most widely used cognitive assessment tools for AD. It contains the following sub-items: word recall task, naming objects and fingers, following commands, constructional praxis, ideational praxis, orientation, word-recognition task, recall of test instructions, comprehension of spoken language, word-finding difficulty in spontaneous speech, and spoken language ability. Our study calculated the correlations of the changes in volume of gray matter atrophy and glucose metabolism in key brain regions in MCI with the ADAS-cog sub-item scores.

### Magnetic Resonance Imaging Scanning

The dataset in the experiment is standard T1- weighted MR images using volumetric 3D Magnetization Prepared-Rapid Gradient Echo (MPRAGE) imaging. The MCI data comes from 25 research centers in China, using 3T scanners from Siemens, GE and Philips. FOV = 256 mm × 256 mm, layer thickness = 1 mm, layers = 170, TE = 3 ms, TR = 1,900 ms, TI = 900 ms and flip angle = 9 degree. The HCs data comes from the ADNI database, also scanned by 3T scanners from Siemens, GE and Philips with the same MPRAGE protocol. FOV = 240 mm × 256 mm, layer thickness = 1 mm, layers = 170, TE = 3 ms, TR = 2,300 ms, TI = 900 ms and flip angle = 9 degree. Further scanning details of ADNI are available in^1^.

### Fluoro-Deoxyglucose Positron Emission Tomography Scanning

Whole brain FDG-PET imaging was performed in this study. The HCs data were obtained from the ADNI database, and the MCI data were obtained from 25 research centers in China. All participants fasted for 4–6 h before the injection of the 18F-FDG PET tracer. Each patient was injected with 0.1 mCi/kg FDG tracer, and scanning began 60 min after tracer injection. The PET scanning time was 15 min (axial FOV = 30 cm, acquisition matrix = 128 × 128, layer thickness = 2.5 mm, layers = 80).

### Image Pre-processing

Statistical Parametric Mapping software (SPM12; Wellcome Department of Cognitive Neurology, London, United Kingdom) and its toolboxes, computational anatomy toolbox (CAT12) and PET partial volume effects 12 (PETPVE12), were used to preprocess MRI and FDG-PET scans. The specific steps are as follows: First, the PET images were co-registered with the original MRI space of the corresponding individual. Second, a voxel-based correction method was used for partial volume correction of the co-registered PET images using PETPVE12. The MR image wasincorporated into the standard Montreal Neurological Institute space and modulated using the Jacobian determinant. Third, the MRI image was segmented, and the deformation field, aligned with the specific template, was obtained using Diffeomorphic Anatomical Registration Through Exponentiated Lie Algebra (DARTEL). The corrected PET images were spatially normalized using the deformation fields obtained at the segmentation step of MRI. Next, in the PET images, the standardized uptake value ratio (SUVR) was generated using the average standardized uptake value of the cerebellar gray matter as the benchmark. Finally, the resulting gray matter images and glucose metabolism images were smoothed using an 8 mm isotropic Gaussian kernel.

### Construction of Brain Networks

A brain network is composed of defined nodes and edges that connect nodes. In this study, an undirected weighted similarity network is established, and the weight of edges represents the correlation of glucose metabolism or gray matter volume between nodes. The gray matter volume determined using MRI and SUVR from FDG-PET images of the 137 cases in the MCI group and 80 cases in the HC group was used to construct the structural and metabolic brain networks. Using the MATLAB (Mathworks Inc., Natick, MA, United States) script, theAnatomical Automatic Labeling (AAL) template was used to divide brain regions into 90 brain regions (Desikan et al., 2006).

The partial Pearson correlation was used to calculate the correlation coefficient between the parameters of each node and the edge of the connecting nodes, considering the effect of age and sex on the edge of the network.

### Calculation of Network Metrics

In this study, the sparsity method was used to threshold the network. Within the range of 5–50%, the network topology parameters under different sparsity thresholds were calculated with 5% step size, and parameters were compared between groups. We chose a network sparsity of 30% to show the network results. Under this sparsity, the network attributes were relatively stable, and the number of nodes in the four brain networks was approximately 90. The figures of correlation matrix of 90 × 90 ROIs for each group have been provided in Supplementary Material. The betweenness centrality of the nodes was considered in the networks. The betweenness centrality of a node i (*BC*_*i*_) is defined as the number of shortest paths between any two nodes that run through the node i. Hub nodes in this study were defined as nodes in which the betweenness centrality was twice the average betweenness centrality of the network. The nodes in which betweenness centrality changed significantly in both structural and metabolic brain networks were defined as key brain regions associated with the pathology of MCI. For the definition of small world, *L*, *C*, *E*_*loc*_, and *E*_*glob*_, see Supplementary Material.

### Statistical Analysis

The permutation test was used to test the statistical significance of group differences in the network parameters. In our study, we obtained a new reference distribution after repeatedly rearranging the observed network data obtained from the HC and MCI groups, calculated the differences between the new groups, and repeated this process 1,000 times. Repeated differences were also recorded. If the differences in the observed networks were contained without 95% of the supposed differences, we accepted that there were significant differences between the two groups.

We also performed a partial correlation analysis to investigate the correlations of mean glucose intake and gray matter volume in key brain regions with individuals’ ADAS-cog scores, adjusted for age and sex with a Bonferroni-adjusted *p* value of 0.016.

## Results

### Participants

As shown in Table 1, the mean age of patients in the MCI group was 66.5 ± 7.7 years, and of individuals in the HC group was 67.3 ± 4.0 years (*p* = 0.441). The sex ratio (M/F) in the MCI group was 61/76 and in the HC group was 35/45 (*p* = 0.912).

**TABLE 1:** Comparison of the structural and metabolic brain networks in patients with MCI and HCs.

	MCI (*n* = 137)	HC (*n* = 80)	*p* value
Age (years)	66.5 ± 7.7	67.3 ± 4.0	0.441
Gender (M/F)	61/76	35/45	0.912
MMSE	24.23 ± 1.69	29.16 ± 1.08	0

*Age and MMSE scoreare described in terms of mean ± standard deviation.*

*P < 0.05 indicates that the difference is significant.*

*MCI, mild cognitive impairment; HC, healthy controls.*

### Structural and Metabolic Brain Networks in Mild Cognitive Impairment and Healthy Control

#### Structural Brain Network Relative to Metabolic Brain Network in Mild Cognitive Impairment

In this study, 30% sparsity was selected to compare the relevant parameters of brain networks, and the weighted matrix constructed under this sparsity is shown in the Supplementary Material. Both the structural and metabolic brain networks in patients with MCI had small-world attributes, and the small-world attribute (σ) of the structural brain network was relatively stronger (σ of the structural brain network = 8.002, σ of the metabolic brain network = 4.375). Using the permutation test, we compared the *L*, *C*, *E*_*loc*_, and *E*_*glob*_ of the two networks. As shown in Table 2, the *L* of the MCI structural brain network was significantly smaller than that of the metabolic brain network (*p* < 0.0001), while the *E*_*loc*_, and *E*_*glob*_ of the structural brain network were significantly larger than those of the metabolic brain network (*p* < 0.0001).

**TABLE 2:** Comparison of the structural and metabolic brain networks in patients with MCI and HCs.

Network parameters	MCI structural brain network vs. MCI metabolic brain network			MCI structural brain network vs. HC structural brain network			MCI metabolic brain network vs. HC metabolic brain network		
	MCI structural network	MCI metabolic network	*p* value	MCI	HC	*p* value	MCI	HC	*p* value
Characteristic path length	2.429	3.211	<0.0001	2.429	2.276	0.01	3.211	2.832	0.047
Clustering coefficient	0.735	0.539	<0.0001	0.735	0.692	<0.0001	0.539	0.597	0.073
Local efficiency	0.510	0.403	<0.0001	0.510	0.535	<0.0001	0.403	0.437	0.123
Global efficiency	0.510	0.404	<0.001	0.510	0.536	<0.0001	0.404	0.437	0.131

*The permutation test was used for all comparisons and a value of *p* < 0.05 indicated that the difference was significant.*

*MCI, mild cognitive impairment; HC, healthy controls.*

#### Mild Cognitive Impairment Relative to Healthy Control in Structural Brain Network

The structural brain networks of both patients with MCI and HCs had small-world properties, and the small-world parameters were almost the same at 30% sparsity (σ of the structural brain network in patients with MCI = 8.002, σ of the structural brain network in HCs = 7.954). We also compared the *L*, *C*, *E*_*loc*_, and *E*_*glob*_ of the two networks using the permutation test. As shown in Table 2, the *L* (*p* = 0.001) and *C* (*p* < 0.0001) of the MCI structural brain network were significantly larger than those of the HC structural brain network, while the *E*_*glob*_ and *E*_*loc*_ of the MCI structural brain network were significantly smaller than that of the HC structural brain network (*p* < 0.0001).

#### Mild Cognitive Impairment Relative to Healthy Control in Metabolic Brain Network

At 30% sparsity, the metabolic brain networks of patients with MCI and HCs had small-world attributes, and the small-world parameters of the MCI metabolic brain network were smaller than the HC metabolic brain network (σ of the metabolic brain network in patients with MCI = 4.375, σ of the metabolic brain network in HC = 5.500). Using the permutation test, we compared the *L*, *C*, *E*_*loc*_, and *E*_*glob*_ of the two networks. Table 2 shows that the *L* of the MCI metabolic brain network was significantly larger than that of the HC metabolic brain network (*p* = 0.047), but there was no significant difference in *C*, *E*_*loc*_, and *E*_*glob*_.

### Screening the Key Areas of Brain Function in Patients With Mild Cognitive Impairment and Hub Analysis

**FIGURE 1:**
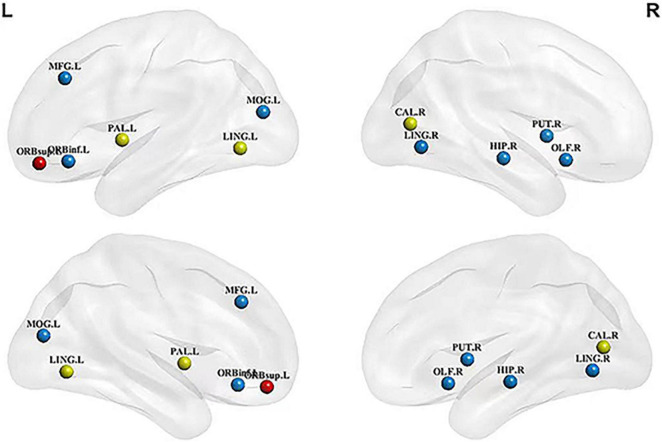
Hubs with significant changes in structural and metabolic networks in MCI and HC groups. The blue sphere represents the hub that is significant only in the structural brain network, red sphere represents the hub that is significant only in the metabolic brain network, and yellow sphere represents the hub that is significant in both networks. There are the key brain regions related to MCI pathology. MCI, mild cognitive impairment; HC, healthy control;LING.L, left lingual gyrus; LING.R, right lingual gyrus; MFG.L, left middle frontal gyrus; ORBinf.L, left orbital inferior frontal gyrus; ORBsup.L, left orbital superior frontal gyrus; PAL.L, left globus pallidus; MOG.L, left middle occipital gyrus; CAL.R, right calcarine fissure and surrounding cortex; HIP.R, right hippocampus; PET.R, right putamen; OLF.R, right olfactory cortex.

The node information for all the hubs in the four networks is shown in Table 3. The betweenness centralities of 10 hubs were significantly different between the HC structural brain network and the MCI structural brain network. These brain regions were located in the frontal lobe, occipital lobe, marginal lobe, and gray matter nucleus (Figure 1). The betweenness centralities of the right putamen, right lingual gyrus, left middle occipital gyrus, left orbital inferior frontal gyrus, right olfactory cortex, and left globus pallidus increased significantly in the MCI structural brain network than in the HC structural brain network, and the increase ranged from 1.018 to 3.035 times. The subsequences from high to low in increased betweenness centrality were the right lingual gyrus, left middle occipital gyrus, right putamen, left inferior frontal gyrus, right olfactory cortex, and globus pallidus, among which the increases in the left inferior frontal gyrus, right olfactory cortex, and globus pallidus in the left orbital region were the same and the smallest. The betweenness centrality of the left lingual gyrus, right hippocampus, left middle frontal gyrus, right talus fissure, and surrounding cortex decreased significantly, with a range of 0.203 to 0.511 times. The subsequences from high to low in decreased betweenness centrality were the right hippocampus, left middle frontal gyrus, left lingual gyrus, and right calcarine fissure and its surrounding cortex.

**TABLE 3:** Structural and metabolic brain network hub nodes in MCI and HC groups.

Hub nodes			*BC*_*i*_ in structural networks			*BC*_*i*_ in metabolic networks		
			MCI	HC	*p* value	MCI	HC	*p* value
Common hub of three networks	MCI structural brain network and HC and MCI metabolic brain networks	INS.L	0.048	0.023	0.154	0.034	0.056	0.907
Common hub of two networks	HC and MCI structural brain networks	LING.L	0.035	0.081	0.018*	0.011	0.022	0.031^▲^
		MTG.R	0.036	0.047	0.427	0.011	0.011	0.367
		MOG.L	0.128	0.045	0.002*	0.011	0.011	0.382
	MCI structural and metabolic brain networks	PUT.R	0.034	0.023	0.018*	0.034	0.012	0.104
	HC metabolic and MCI structural brain networks	LING.R	0.035	0.012	0.019*	0.011	0.045	1.000
Unique hub	MCI structural brain network	STG.L	0.056	0.012	0.092	0.011	0.023	0.929
		STG.R	0.037	0.011	0.107	0.012	0.011	0.229
		MTG.L	0.036	0.011	0.179	0.011	0.012	0.514
	HC structural brain network	HIP.R	0.012	0.057	0*	0.011	0.011	0.217
		MFG.L	0.017	0.050	0.006*	0.015	0.011	0.851
	MCI metabolic brain network	ORBinf.L	0.012	0.011	0*	0.037	0.023	0.116
		OLF.R	0.012	0.011	0*	0.034	0.024	0.390
		PAL.L	0.012	0.011	0*	0.034	0.011	0.014^▲^
	HC metabolic brain network	CAL.R	0.012	0.023	0.011*	0.011	0.034	0.015^▲^
		ORBsup.L	0.012	0.023	0.183	0.014	0.030	0.014^▲^

*The table summarizes all the hub nodes of the structural and metabolic brain networks in the MCI and HC groups.*

*Significant difference between the structural networks in the HC and MCI groups (*p* < 0.05), ^▲^Significant difference between the metabolic networks in the HC and MCI groups (*p* < 0.05).

*MCI, mild cognitive impairment; HC, healthy controls; INS.L, left insula; LING.L, left lingual gyrus; MTG.R, right middle temporal gyrus; MOG.L, left middle occipital gyrus; PET.R, right putamen; LING.R, right lingual gyrus; STG.L, left superior temporal gyrus; STG.R, right superior temporal gyrus; MTG.L, left middle temporal gyrus; HIP.R, right hippocampus; MFG.L, left middle frontal gyrus; ORBinf.L, left orbital inferior frontal gyrus; OLF.R, right olfactory cortex; PAL.L, left globus pallidus; CAL.R, right calcarine fissure and surrounding cortex; ORBsup.L, left orbital superior frontal gyrus.*

Compared with the HC metabolic brain network, four hubs were significantly altered in the MCI metabolic brain network. These brain regions were distributed in the frontal and occipital lobes (Figure 1). Among them, the betweenness centrality of the left globus pallidus was significantly increased (2.947 times that of the HC group). The betweenness centralities of the left lingual gyrus, right calcarine fissure and its surrounding cortex, and left superior frontal gyrus of the orbital region were significantly decreased, with a range of 0.330 to 0.500 times. The subsequences from high to low in decreased betweenness centrality were the right calcarine fissure and its surrounding cortex, left superior frontal gyrus, and left lingual gyrus.

**FIGURE 2:**
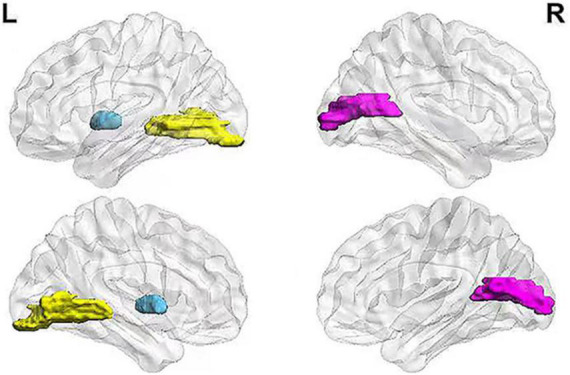
Key brain regions related to MCI pathology. The figure shows the selected key brain regions associated with MCI pathology. The blue area represents the left globus pallidus, yellow area represents the left lingual gyrus, and pink area represents the right calcarine fissure and its surrounding cortex. MCI, mild cognitive impairment.

The key brain regions screened from all hub nodes were shown in the left lingual gyrus, left globus, right calcarine fissure, and its surrounding cortex. The betweenness centralities of patients in the MCI group showed significant changes in brain networks obtained from both MRI and FDG-PET scans (Figure 2). The betweenness centralities of the left globus pallidus in both structural and metabolic brain networks were increased in the MCI group (1.018 times and 2.947 times of the HC group, respectively), while those of the right calcarine and its surrounding cortex and left lingual gyrus in both structural and metabolic brain networks was decreased in the MCI group (0.511 times and 0.330 times of the HC group in the right calcarine and 0.434 times and 0.500 times of the HC group in the left lingual gyrus, respectively).

### Correlation Analysis Between Key Brain Regions and Cognitive Function in Mild Cognitive Impairment

**FIGURE 3:**
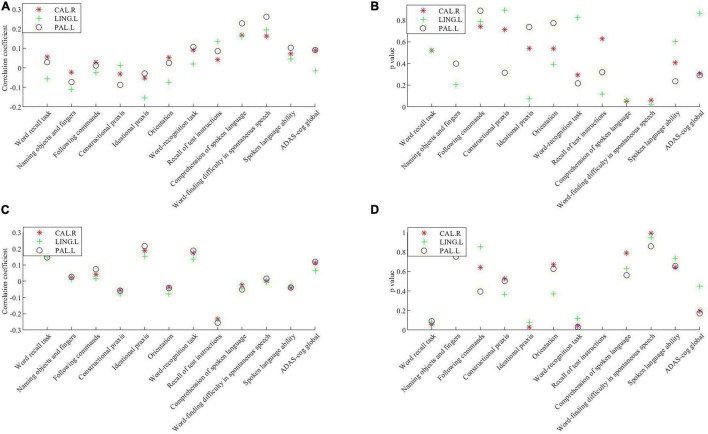
Correlations between the volume of the gray matter atrophy/the mean glucose intake in key brain regions and cognitive scores. **(A)** Correlation coefficient between the volume of gray matter atrophy in key brain regions and cognitive scores. **(B)** *P* value of the correlations between the volume of gray matter atrophyin key brain regions and cognitive scores. **(C)** Correlation coefficient between the mean glucose intake in key brain regions and cognitive scores. **(D)** *P* value of the correlations between the mean glucose intake in key brain regions and cognitive scores. Significance was determined with a Bonferroni adjusted *p* value of 0.016. CAL.R, right calcarine fissure and surrounding cortex; LING.L, left lingual gyrus; PAL.L, left globus pallidus; ADAS-cog, Alzheimer’s Disease Assessment Scale-Cognitive.

Partial correlations between key brain regions and cognitive scores are shown in Figure 3. The volume of gray matter atrophy in the left globus pallidus was positively correlated with the comprehension of spoken language (*p* = 0.024, corrected for multiple comparisons) and word-finding difficulty in spontaneous speech item scores (*p* = 0.007, corrected for multiple comparisons) in the ADAS-cog, but was not significantly correlated with other sub-items and total score items. The three key brain regions were the left glossal gyrus, left globus pallidus, and right talus cleft and its surrounding cortex, and their mean glucose intakes were significantly negatively correlated with the instruction items of the remembering test in the ADAS-cog (*p* = 0.020, *p* = 0.014, and *p* = 0.008, respectively, corrected for multiple comparisons). The mean glucose intake of the left globus pallidus was significantly positively correlated with the ideational praxis in ADAS-cog, with a low correlation coefficient.

## Discussion

This study reported that the changes in network topology parameters or hub levels of the structural brain network are more than those in the metabolic brain network in MCI. To the best of our knowledge, this is the first study to present the differences among multimodal brain networks constructed using MRI and FDG-PET data of patients with MCI. The right calcarine, left lingual, and left globus pallidus were identified as the key brain regions associated with cognitive function.

Our research showed that the topological properties of structural and metabolic brain networks differed between patients with MCI and HCs. Similar results have been reported by few single-mode brain network studies that collected data from MRI, electroencephalogram, and fMRI of patients with MCI (Stam et al., 2007; Sanz-Arigita et al., 2010; Phillips et al., 2015). The contrasting changes in the parameter intensity of two brain networks between patients with MCI and HCs were highlighted in this research. The structural brain network showed stronger small-world attribute, shorter *L*, higher *C*, lower *E*_*loc*_, and *E*_*glob*_ than metabolic brain networks. A longer *L* reflects a decrease in remote connection capability, and a higher *C* reflects the strengthening of local connections (Yao et al., 2010). These results suggest that structural and metabolic brain networks are distinct in their internal structures and information transmission modes.

Based on the comparison of the number of changing parameters and hubs, we found differences in the topological properties of brain networks between MRI and FDG-PET in MCI. This research showed that there were four topological parameters in the structural brain networks and only one in the metabolic brain network. The *L*, *C*, *E*_*loc*_, and *E*_*glob*_ of the structural brain network differed between the MCI group and the HC group, while only *L* was significantly greater in the metabolic brain network of HCs than of patients with MCI. These findings show that damage to the structural brain network may be larger than that to the metabolic brain network in the MCI disease phase. This finding is similar to that of a previous study, which was based on different construct networks obtained using DTI data and metabolic networks obtained using fMRI data. The study showed that in the structural network, patients with MCI showed lower *E*_*loc*_ and *C* than HCs, while no significant parameter changes were observed in the functional brain network (Filippi et al., 2020). Although the used data were different for constructing the structural and metabolic network connections, two studies showed consist results that the damage in the parameters of the structural brain network was greater than that in the parameters of the metabolic brain network in patients with MCI. In addition, a comparison of the number of hubs in the two brain networks showed that the severity of harm in the structural network is greater than in the metabolic network. As an important reflection of the high betweenness centrality of nodes in the brain network, a hub point was used to reflect the critical position of nodes in brain networks, which could be a more focused representation of changes in brain networks. In our survey, ten hubs showed significant changes in the structural brain network, while only four showed significant changes in the metabolic brain network. Our research demonstrated that structural network changes were more than the metabolic network changes at the hub level in MCI. The possible reasons for the inequality between the two networks could be the damage to the structural brain network anterior to the metabolic network. This possibility shows a certain degree of coincidence with the hypothesis proposed in a previous study, in which the patterns of functional connectivity in the brain were proposed to be determined by, but not limited to, structure (Palesi et al., 2016). In our research, the shorter side length and *C* of the *L* in the MCI structural brain network reflected a decrease in distant connection ability and an increase in local connection. We believe that this is an automatic compensation mechanism within the brain structure network. After the structural network is damaged, the internal topological properties are adjusted to retain the relatively stable parameters in the metabolic network. However, the accuracy of this conclusion needs to be verified through multi-mode brain network studies.

We obtained the key brain regions through scanning of the hubs, and the betweenness centrality of the hubs varied significantly between the MCI and HC groups in both structural and metabolic brain networks. In this study, we identified the left lingual gyrus, right talus cleft and its surrounding cortex, and left globus pallidus as the three key brain regions. The left lingual gyrus and the right talus cleft and its surrounding cortex were significantly reduced in the two brain networks, while the left globus pallidus was significantly increased and played a certain compensatory role in the flow of network information. We also investigated the association of the three key brain regions with cognitive function evaluated using ADAS-cog. We found that the volume of gray matter atrophy in the left globus pallidus was significantly positively correlated with comprehension of spoken language and word-finding difficulty in spontaneous speech item scores in ADAS-cog, while the glucose intake in the three key brain regions remained significantly negatively correlated with remembering test instructions items in ADAS-cog, indicating that these three key brain regions were involved in cognitive function. In previous studies, the three key brain regions have been reported to be associated with cognition. Schmidt et al. (2007) used fMRI to explore the brain mechanisms of viewpoint change in 3D spatial visual memory tasks and found that the left lingual gyrus plays a special role in the coding of spatial scene memory and center. Zhang et al. (2001) applied the echo plane technique for fMRI of blood oxygen-level dependence of the human visual cortex under two contrasting conditions of stimulation and rest and found that the lesions of the talar fiscus, which is located above the lingual gyrus and the hippocampus, may cause displacement of the visual cortex in patients with AD. The lesions of the globus pallidus may be caused by indirect dementia and cognitive dysfunction (Kim et al., 2008).

The correlation analyses performed in this study showed that the globus pallidus was related to two ADAS-cog sub-items in the structural network presented by the volume of gray matter atrophy, but only one sub-item in the metabolic network was constructed according to the value of glucose intake. In addition, both the left lingual gyrus and the right talus cleft and its surrounding cortex in the structural network were significantly correlated with one sub-item, but no significant correlation with any of the sub-items in the metabolic network was evident. We consider that the different correlations between the same brain region and sub-items in different modal images may be due to their varying roles in structural or metabolic brain networks. Interestingly, there was a significant positive correlation between glucose intake in the left globus pallidus and ideational praxis in the ADAS-cog. A possible reason could be the functional adaptation or compensation of pathology-induced injury in the course of disease change, and related mechanisms need to be discussed in future studies.

Our study is the first to report the differences in topological properties of two brain networks obtained using MRI and FDG-PET data in MCI, but has some limitations. This was a cross-sectional study. Longitudinal studies are necessary to assess the changes in brain networks during the course of the disease. The topological properties of brain networks depend on the construction methods of the networks. However, there is no consensus on the research methods for networks. In addition, all networks built in this study were group networks; therefore, the analysis of individual networks should be considered at a later stage. The HC group of this study was obtained from the ADNI database, and the results of this study should be further verified by expanding and diversifying the sample at a later stage.Moreover, in the light of the limitations of multiple comparisons, our findings should be regarded as preliminary.

In this study, we constructed cluster networks through MRI and PDG-PET images of MCI and HC groups respectively, and compared the differences in the number of topological parameter changesas well as the differences in the number of central nodes between structural and metabolic networks. In addition, we found that the betweenness centrality of the right calcarine fissure and its surrounding cortex, left lingual gyrus, and left globus pallidusdiffered significantly between HCs and patients with MCI in both structural and metabolic networks, and both structural and metabolic brain networks were related to cognition. Our results indicate that the structural network changeslarger than the metabolic network during MCI stage, which helps us better understand the network changes during the pathogenesis of AD. Our findings highlight the important role of the construction of a multimodal brain networkin identifying key brain regions of MCI and provide insights into the use of hubs to describe the transmission of in the brain.

## Data Availability Statement

The original contributions presented in the study are included in the article/Supplementary Material, further inquiries can be directed to the corresponding author/s.

## Ethics Statement

The studies involving human participants were reviewed and approved by Ethics Committee of Xuan Wu Hospital (No. 2017-058). The patients/participants provided their written informed consent to participate in this study.

## Author Contributions

XL, YC, YY, and WW collected the patients’ clinical data. WZ and XK completed the data statistics. CW, SG, QZ, and WZ drafted the manuscript. CW designed the research project and revised the manuscript with BS. LJ and JL participated in the case diagnosis. All authors read and approved the final manuscript.

## Conflict of Interest

The authors declare that the research was conducted in the absence of any commercial or financial relationships that could be construed as a potential conflict of interest.

## Publisher’s Note

All claims expressed in this article are solely those of the authors and do not necessarily represent those of their affiliated organizations, or those of the publisher, the editors and the reviewers. Any product that may be evaluated in this article, or claim that may be made by its manufacturer, is not guaranteed or endorsed by the publisher.

## Footnotes

1
http://adni.loni.usc.edu/
