# Supplementary material for: A Comparative Study of Structural and Metabolic Brain Networks in Patients With Mild Cognitive Impairment
Source: Front Aging Neurosci. 2021 Dec 6;13:774607. doi: 10.3389/fnagi.2021.774607 (PMC8687449; doi:10.3389/fnagi.2021.774607)
Supplement: Supplementary file 1 [file Data_Sheet_1.PDF]

Supplementary Material

Supplementary Data

The calculation formula of each parameter in this study.

The characteristic path length. For a binary network, the shortest path is the path with the least number of edges connecting two nodes, and the number of edges in the path is the shortest path length between the two nodes. It describes the best path from one node in the network to another node. Through the shortest path, information can be transmitted faster, thus saving system resources. However, for the calculation of the shortest path length of similar weighted network, it is necessary to take the weight value to a number, define the shortest path as the reciprocal sum of the weight between two nodes, and the reciprocal sum of the shortest path as the shortest path length. For example, node i and node j are connected by two edges with weights of w_{ik} and w_{kj} , then the path length between these two nodes is calculated by the following equation:

$$l_{ij} = \frac{1}{\frac{1}{w_{ik}} + \frac{1}{w_{kj}}}$$

Taking the shortest path length between any two nodes in the network as an average, the characteristic path length L of the entire network can be obtained:

$$L = \frac{1}{N(N-1)} \sum_{i,j \in V, i \neq j} l_{ij}$$

Where l_{ij} represents the shortest path length between node i and node j, and N represents the total number of nodes.

Network clustering coefficient. The clustering coefficient C_i of node i describes the possibility that the neighbors of node i are neighbors to each other. The two nodes with edges between the two nodes are neighbors to each other. The larger the node clustering coefficient is, the closer the connection with the neighboring points is, and the stronger the local information transmission capacity is. The network clustering coefficient is the mean of all node clustering coefficients. The clustering coefficient of each node is calculated according to the definition of clustering coefficient by Petter Holme, and the calculation formula is as follows:

$$C_i = \frac{\sum_{j,k} w_{ij} w_{jk} w_{ki}}{\max_{ij} w_{ij} \sum_{j,k} w_{ij} w_{ik}}$$

Where w_{ij} represents the weight of edges between nodes i and j.

Network local efficiency. Both the network local efficiency and clustering coefficient can describe the network local information transmission capacity and reflect the network's ability to resist random attacks. The larger the clustering coefficient and local efficiency, the stronger the ability of local information transmission and attack resistance. The calculation of node i is as follows:

$$E_i = \frac{1}{N_{G_i}\left(N_{G_i} - 1\right)} \sum_{j \neq k \in G_i} \frac{1}{l_{j,k}}$$

Where G_i is the subgraph formed by the neighbors of node i, N_{G_i} is the number of nodes of the subgraph, and $l_{j,k}$ is the length of the shortest path between node j and node k. The local efficiency of the network can be obtained by taking the average value of the local efficiency of each node. The calculation is as follows:

$$E_{loc} = \frac{1}{N} \sum_{i \in V} E(i)$$

Global efficiency. The parameters of global efficiency and characteristic path length quantify the global information transmission capacity. The shorter the characteristic path length is, the higher the global efficiency is, and the stronger the global information transmission capacity is. The calculation formula is as follows:

$$E_{glob} = \frac{1}{N(N-1)} \sum_{i,j \in V, i \neq j} \frac{1}{l_{ij}}$$

Small world parameters. Small-world networks, which are between regular networks and random networks, have high clustering coefficient and short feature path length. The small-world parameter σ is used to describe the strength of the small-world attribute of the network. $\sigma > 1$ indicates that the network has small-world attribute. The larger the σ is, the stronger the small-world attribute will be. The small-world parameter calculation formula is as follows:

$$\sigma = \frac{\gamma}{\lambda}$$

Where $\gamma = \frac{C_{\text{real}}}{C_{\text{random}}} >> 1$， $\lambda = \frac{L_{real}}{L_{random}} \approx 1$. The subscript real denotes a computational network, and random denotes a random network.

Betweenness centrality. The betweenness centrality of a node is defined from the perspective of information flow. The more the number of shortest paths passing through a node, the greater the betweenness centrality of the node. The betweenness centrality of a node is the quotient of the number of shortest paths through the node divided by the total number of shortest paths in the network. The calculation formula is as follows:

$$N_{bc}(i) = \sum_{j \neq i \neq k \in G} \frac{\alpha_{jk}(i)}{\alpha_{jk}}$$

Where, $\alpha_{jk}(i)$ represents the number of shortest paths between node j and k that passing through node i, and α_{jk} represents the number of all shortest paths between node j and node k.

Supplementary Figure

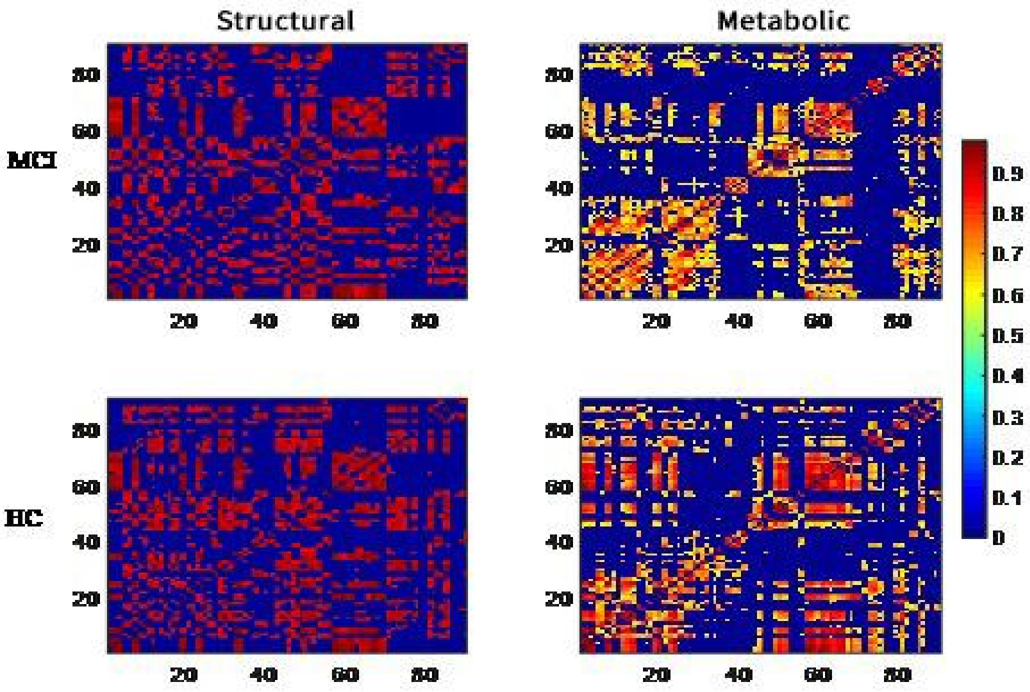

Note. Brain networks at 30% sparsity. The horizontal and vertical coordinates represent 90 nodes of brain regions corresponding to AAL. The upper left corner is MCI structural brain network, the lower left corner is HC structural brain network, the upper right corner is MCI metabolic brain network, and the lower right corner is HC metabolic brain network.

AAL, Anatomical Automatic Labeling; MCI, mild cognitive impairment; HC, healthy controls
